# Rave gone wrong: MDMA- induced medical emergency at electrical daisy carnival. A case report^☆^

**DOI:** 10.1016/j.toxrep.2024.101739

**Published:** 2024-09-20

**Authors:** Charissa Alo, Mutsumi J. Kioka

**Affiliations:** a Kirk Kerkorian School of Medicine at UNLV, Las Vegas 89106, USA; b Kirk Kerkorian School of Medicine at UNLV, Department of Internal Medicine, 625 Shadow Lane, Las Vegas 89106, USA

**Keywords:** MDMA, overdose, multi-organ failure, intensive care unit, respiratory failure, coagulopathy, renal failure, stroke

## Abstract

3, 4-methylenedioxyamphetamine (MDMA) has gained significance over the years, especially at rave festivals, as a recreational drug for its noted effects in mood enhancement and autonomic stimulation. While these effects have been noted, severe adverse outcomes, and even death, following the ingestion of MDMA have been recorded.

We present a 35-year-old male who ingested the drug at the Electric Daisy Carnival (EDC), the largest electronic dance music festival in North America as of 2024 [1]. Every year, many young adults are brought to local hospitals from the festival for drug overdoses, hyperthermia, and dehydration. At the festival, the patient was witnessed to have a seizure, presented with altered mental status and deemed hyperthermic at 109 degrees Fahrenheit. For these reasons, he was rapidly intubated and submerged in an ice bath at the festival’s medical tent. At the county hospital, the patient was diagnosed with multiorgan failure, cerebrovascular ischemia, and coagulopathy. He received life-saving treatment such as continuous renal replacement therapy as well as intubation for acute hypoxemic respiratory failure. MRI of the brain showed central- embolic infarcts and the patient was closely monitored in the intensive care unit (ICU) for eight days. After twenty days of inpatient treatment, the patient was discharged. He was discharged with his mental status at baseline and without gross neurologic deficits. A permacath was placed for hemodialysis to be continued outpatient.

This case report highlights the importance of prompt medical management which can be crucial for patient survival following a life-threatening overdose with MDMA. It also exemplifies the need for increasing social awareness regarding the severe and detrimental outcomes an MDMA overdose can cause as this drug continues to be widely used in the setting of rave and music festivals.

## Highlights

• MDMA toxicity can present with life- threatening symptoms such as multi- organ failure.
• The acute management of MDMA toxicity is crucial as it can affect multiple organ systems.
• The life- threatening side- effects of MDMA toxicity should be further addressed as the popularity of this party drug continues to be prevalent.

## Graphical Abstract

**Figure:**
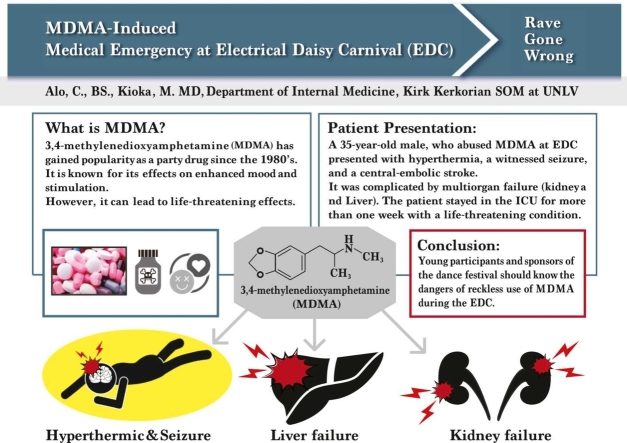

## 1 Introduction

Since the 1980’s, 3, 4-methylenedioxyamphetamine (MDMA), known as ecstasy, has become a popular recreational psychostimulant particularly used at music or rave festivals. Notoriously utilized in these settings, MDMA is known to have stimulating effects on the autonomic nervous system as well as reported enhancements in mood contributing towards its trending rise in recreational usage [1], [2], [3], [4], [5], [6]. Users have reported increased effects of euphoria and increased levels of energy which can be attributed to the drug’s ability to indirectly increase serotonin, dopamine, and norepinephrine levels [7], [8]. In addition, it has been noticed that consumption of MDMA can result in an increased perception of intimacy and closeness. These notions have been suggested to be due to the drug’s ability to increase prolactin secretion mimicking a post-orgasmic state [9]. These psychostimulant enhancements contribute to the popularity of this drug in environments such as rave festivals which create extensive and elaborate visuals utilizing technology attracting large crowds of individuals at each event.

MDMA’s acute effects on the Central Nervous System (CNS) as well as potential long-term effects have been previously studied since the emergence of this drug. MDMA’s ability to cause enhancements in mood is suggested to be due to its ability to indirectly increase the release of serotonin and block the reuptake of the molecule. This has been theorized by MDMA’s ability to block tryptophan hydroxylase, the rate-limiting enzyme in the synthesis of serotonin [4], [10]. This reaction leads to the indirect upsurge of serotonin but may also lead to life-threatening toxic outcomes such as serotonin syndrome. In addition, MDMA may also pose a threat to serotonin receptor ligands as previous findings suggest more aggressive uses of MDMA can lessen availability of global and regional serotonin (5-HT) receptor ligands [11]. The ability of MDMA to alter the autonomic nervous system may temporarily cause a positive experience for the user, but its potential to cause severely detrimental side effects may not always be considered in the setting of its recreational usage.

Side effects, and even death, have been studied and reported throughout the years. Common, and non-permanent, side effects include bruxism and tachycardia [7]. However, severe adverse effects have been previously identified through literature and case reports such as hyperpyrexia, rhabdomyolysis, multi-organ failure, and disseminated intravascular coagulopathy (DIC) [7], [12]. Severely elevated body temperatures have been suggested to be due to multiple etiologies in the setting of an MDMA overdose such as the drug and its metabolites’ effect on CNS temperature regulation, the constriction of skin vessels, and increased muscle activity in the setting of a crowded music festival [4]. The resulting hyperthermia can thus contribute to rhabdomyolysis and acute kidney injury, two commonly known life-threatening outcomes. Furthermore, the use of MDMA has also been associated with cerebrovascular accidents proposed by the possibility of MDMA causing arterial vasospasm [13], [14]. These well-known side effects can cause great harm and have been known to cause death among potential overdoses [3].

This case report discusses the clinical presentation and recovery of a patient who was diagnosed with cerebral infarctions, multiorgan failure, and coagulopathy following the ingestion of MDMA at a rave festival. This case report highlights MDMA’s capacity to simultaneously affect various organ systems including the central nervous system, the lungs, the liver, and the kidney. The effect of multiple organ systems prompted medical care that included a primary care team and specialists from nephrology and neurology. While the effects of MDMA toxicity have been previously reported, this case report emphasizes the importance of timely management in addressing life-threatening diagnoses that lead to patient survival. In addition, we hope to highlight the necessity of increased public awareness regarding the grave impact of an MDMA overdose.

## 2 Case report

The patient is a 35-year-old male with no known past medical history who presented to the emergency department after being transported from Electric Daisy Carnival. At the rave festival, the patient was brought to the medical tent with altered mental status and hyperthermia at 109 degrees Fahrenheit (F) after he and his partner ingested MDMA. The patient received 200 mg of dantrolene and was quickly placed in an ice bath for approximately twenty to thirty minutes. At the scene of the festival, seizure-like activity was noted by medical personnel, and he was given 5 mg of midazolam. Rapid sequence intubation was performed in the medical tent of the festival and was completed with 100 mg of rocuronium and 200 mg of ketamine. On the way to the county hospital, the patient was hypotensive with systolic blood pressure in the 60 s and diastolic in the 40 s. He was given 4 L of cooled saline and a total of 800 mcg pushes of IV phenylephrine.

During arrival at the emergency department, the patient was hypothermic at 88 F. One liter of warm normal saline as well as 2 liters of lactate ringer was given. The patient continued to be hypotensive with systolic blood pressures ranging in the 90’s and diastolic blood pressures ranging in the 60’s. Twenty micrograms of epinephrine were given, and the patient was started on a norepinephrine drip. Urine toxicology screening confirmed the presence of tetrahydrocannabinol (THC) and amphetamines. Labs of the patient collected at the time of admission showed multi-organ failure that included the central nervous system, lungs, kidneys, and liver.

### 2.1 Central nervous system

**Fig. 1:**
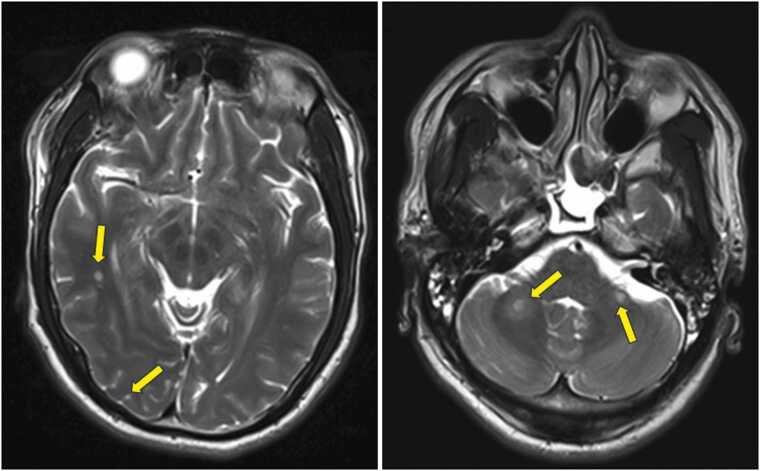
MRI brain showed multiple small foci (yellow arrows) of restricted diffusion, suggestive of central-embolic microinfarcts.

Given that the patient was witnessed to have a seizure at the field of the festival, an MRI of the brain without contrast was performed and showed multiple small foci of restricted diffusion involving the supratentorial and infratentorial brain, suggestive of central-embolic microinfarcts. This is displayed in Fig. 1 showing an infarct in the cerebellar area. To rule out cardiac etiologies, a transesophageal echogram was performed which showed a left atrial appendage free of thrombus. The echocardiogram performed was grossly normal as well. MR angiograms of the head and neck were within normal limits. An EEG was also performed which showed diffused slow tracing consistent with metabolic encephalopathy. The patient was started on Keppra 500 mg twice a day for seizure prophylaxis.

### 2.2 Pulmonary system

**Fig. 2:**
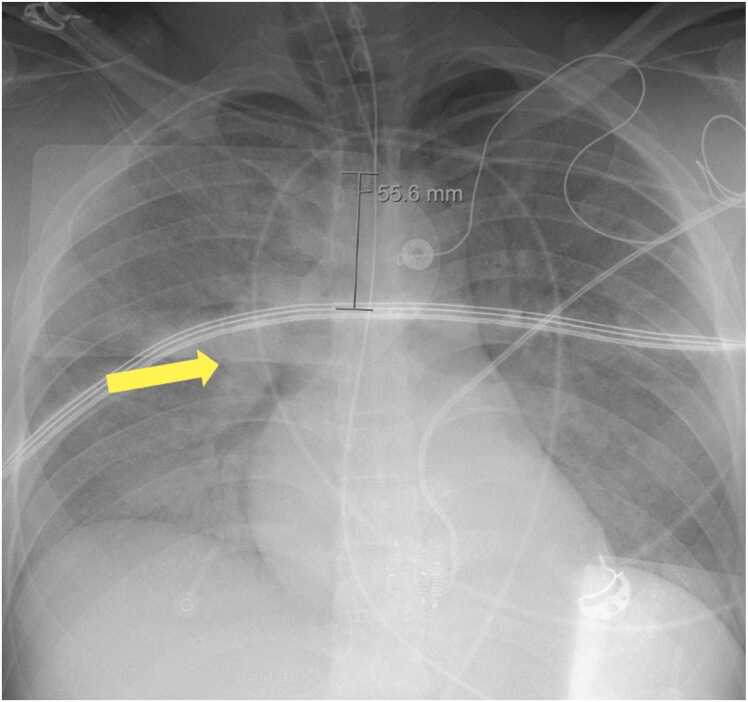
The patient’s chest x-ray on admission showed diffuse opacities throughout the lungs with a consolidation on the right perihilar margin (yellow arrow).

Injury to the lungs was discovered in an initial chest x-ray showing diffuse ground-glass opacities with consolidations in the right perihilar margin seen in Fig. 2. Arterial blood gasses on admission showed metabolic acidemia with a pH of 6.94, carbon dioxide levels at 59.1 mmHg, oxygen levels at 86.0 mmHg, and bicarbonate levels at 12.6 mmol/L. The patient was extubated after three days of hospitalization in the ICU. The patient was then oxygenated with continuous positive airway pressure. He was able to use a nasal cannula after six days in the ICU.

### 2.3 Renal system

Evidence of kidney failure was indicated by an elevated creatine kinase, and creatinine found on admission. Creatine kinase was recorded as 2206 U/L with creatinine measured at 1.88 mg/dL. As mentioned before, the patient initially presented with acidemia with a pH of 6.94. To address the acidemia, the patient was started on a bicarbonate drip. Electrolytes were also replaced as necessary, and repeat creatine kinase levels twenty- four hours after admission were measured at 59,245 U/L. To address the acute renal failure and rhabdomyolysis, the patient was then started

on continuous renal replacement therapy (CRRT) with nephrology closely following his case. He received CRRT for five days and was started on a hemodialysis schedule after. Creatinine kinase peaked at over 100,000 U/L his fourth day of admission. Creatine kinase results during this patient’s hospitalization in the intensive care unit are listed in Table 1.

**Table 1:** Trend of Creatinine Kinase.

Day	1	2	3	4	5	6	7	8
CK (U/L)	2206	59,245	88,772	>100,000	65,801	99,336	24,300	10,403

CK: creatinine Kinase

### 2.4 Hepatic system

The patient was also noted to have injury to the liver shown by elevated transaminase levels. Initial aspartate aminotransferase levels were noted to be 378 IU/L and alanine aminotransferase levels were 560 U/L. These levels were followed and down trended throughout admission which is displayed in Table 2. Liver enzymes peaked at day six.

**Table 2:** Trend of Liver Enzymes.

Day	0	1	4	6	8	11
AST (U/L)	378	2638	1733	1900	606	342
ALT (U/L)	560	1763	1777	1230	641	418

AST: aspartate aminotransferase, ASL: alanine aminotransferase

### 2.5 Hematologic system

Furthermore, evidence of present coagulopathy was noted with fibrinogen levels decreased at 108 mg/ dL and activated partial thromboplastin time was slightly elevated at 32 seconds. Prothrombin time and International Normalized Ratio were also elevated at 16.6 seconds and 1.63 respectively. The patient’s platelet count was also 21,000 k/mm3 on admission.

### 2.6 Clinical resolution

**Fig. 3:**
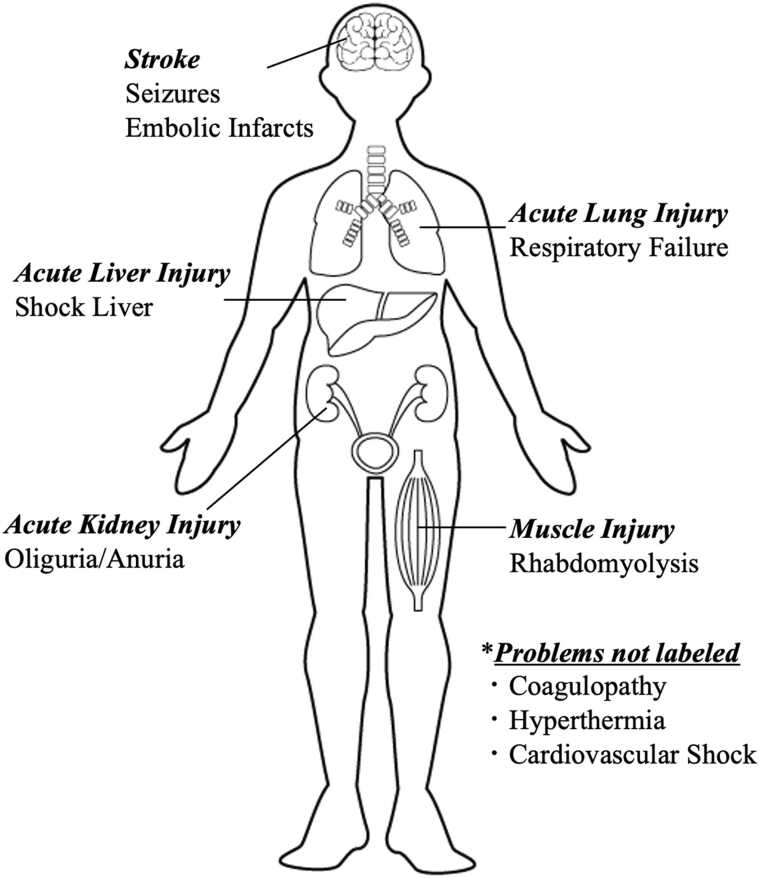
A labeled diagram of the affected organ systems.

After eight days in the ICU, the patient was downgraded to the intermediate care unit. The pertinent findings in the ICU are displayed in Fig. 3. At the time of downgrade, the patient’s mentation and mood were markedly improved. He was able to participate in a conversation and had the capacity for the situation. Neurologic examination showed intact gross cranial nerve function without any focal deficits. The patient was subjectively weak in bilateral upper and lower extremities and received care from physical therapy during his hospitalization. The patient was discharged from the hospital after twenty days. He had a permacath placed before leaving as he will continue with hemodialysis in the outpatient setting and remain oliguric.

## 3 Discussion

This case presentation shows extensive injury to multiple organ systems caused by the ingestion of MDMA. The extent of the patient’s injuries and his survival suggest the significance of timely management. Had he stayed unattended at the rave festival for a longer period of time without medical management, it is uncertain whether he would have survived with the same prognosis.

It is important to note that the patient’s urine drug screening on admission was positive for amphetamines as well as THC. The patient corroborated his ingestion of MDMA at the rave festival but denied taking THC on the evening of the rave festival. Although the patient’s presentation aligns with previous findings of an MDMA overdose, it is important to still acknowledge the presence of THC. Previous literature suggests that recreational users of MDMA often use THC as well, and the drugs combined may have interactive properties rather than additive properties [15]. Although the primary mechanism is still largely unknown, a previous study has demonstrated that the combined use of THC and MDMA altered EEG waves with more significance than the power of each drug used alone [16]. These findings suggest that THC may alter the effects of MDMA, especially in the CNS. However, the significance and clinical correlation of this proposed relationship should be further studied. And while the patient denied THC use during the night of the incident, the presence of THC in his urine drug screen may have indicated present metabolites.

MDMA overdose can have lethal effects that have manifested as multi-organ failure seen in previous literature and case reports. To address MDMA intoxication, promptly addressing potentially lethal effects can vastly affect patient outcomes. In this case, the patient was found hyperthermic at the Electric Daisy Carnival and immediately underwent rapid sequence intubation. MDMA-induced hyperthermia can contribute to rhabdomyolysis, renal failure, acute respiratory distress syndrome, and acidosis [1]. With previous literature suggesting hyperthermia to be a result of direct drug action on the central nervous system, MDMA-induced hyperthermia is notorious for contributing to patient mortality [4], [6], In combination with dehydration and strenuous activity such as dancing in large crowds in a rave festival, these concomitant factors could have definitely contributed towards the patient’s severe hyperthermia at 109 F. Adequate medical resources at the rave festival and prompt transport to the city’s county hospital could have possibly played a role in the patient’s survival as well. It is imperative for music festivals functioning at the level of EDC to be knowledgeable of potential medical emergencies such as this one, and fortunately, the medical staff was equipped and prepared.

Last but not least, the patient’s renal function was significantly impaired following ingestion. With acute kidney injury being a commonly seen feature of MDMA overdose, kidney injury is greatly influenced by rhabdomyolysis [17]. The damage to tissues and accumulation in the kidney tubules is reflected in the patient’s creatinine kinase measuring at over 100,000 U/L during his hospitalization (creatine kinase trends displayed in Table 1). The patient required hemodialysis to be downgraded with his estimated glomerular filtration rate measuring 19 mL/ min/ 1.73 m^2^. While he required hemodialysis to be downgraded, the long-term effects on kidney function should be further investigated and followed after the case of an MDMA overdose as it can greatly affect patient life-quality.

## 4 Conclusion

The adverse effects of MDMA can have life-threatening consequences, and as reflected in this case report, its effects should be managed immediately. From the initial scene of Electric Daisy Carnival to the setting of the intensive care unit, the patient received prompt and preemptive steps that addressed life-threatening problems such as cerebrovascular ischemia, coagulopathy, and multi-organ failure.

Clinicians need to address symptoms of MDMA overdose quickly, but with the rise of its recreational usage, more public awareness should be spread regarding the potentially lethal effects of this psychostimulant. Because the patient’s symptoms started at Electric Daisy Carnival, the presence of a well-equipped medical tent was also an essential feat in the management of this overdose. While this patient was able to survive multiple ailments, the effects this drug can have on his life after discharge may prove to be more permanent such as hemodialysis. Therefore, the organizers of this festival need to recognize the dangers associated with the event and implement further measures to reduce the risk of young people suffering permanent physical harm.

## Declaration of Competing Interest

The authors declare that they have no known competing financial interests or personal relationships that could have appeared to influence the work reported in this paper.

## Data Availability

No data was used for the research described in the article.
